# Tell me your date of birth, and I will tell you how good you are in orienteering

**DOI:** 10.3389/fspor.2025.1558135

**Published:** 2025-03-13

**Authors:** Alberto Ferriz-Valero, Javier Montiel-Bontmatí, Ove Østerlie, Juan Pedro Caraça-Valente, Alberto Mínguez-Viñambres, Héctor Esteve-Ibáñez

**Affiliations:** ^1^ Department of General Didactic and Specific Didactics, Physical Education and Knowledge Advancement (PEAK) Research Group, University of Alicante, Alicante, Spain; ^2^ Department of Teacher Education, Research Group Digital Technology in Physical Education and Sport (DiTePES), Trondheim, Norway; ^3^ Department of General Didactic and Specific Didactics, University of Alicante, Alicante, Spain; ^4^ Department of Teacher Education, Norwegian University of Science and Technology, Trondheim, Norway; ^5^ Department of Computer Languages and Systems and Software Engineering, Polytechnic University of Madrid, Madrid, Spain; ^6^ Counsellor for Education, Science and Universities of Madrid, Madrid, Spain; ^7^ Department of Preparation and Physical Conditioning, Faculty of Physical Activity and Sports Sciences, Catholic University of Valencia “San Vicente Mártir”, Torrent, Spain

**Keywords:** relative age, orienteers, performance, youth, competition, races

## Abstract

Orienteering is a sport where participants must choose the best route between control points marked on the map, combining it with their displacement capacity. It combines endurance running with mental capacity. As in other sports, age can be a determinant in defining differences among youth runners. In this research, the hypothesis is that older orienteers will show better performance than younger orienteers within the same competitive group, for both girls and boys. Overall, official results of the FEDO (Spanish Federation of Orienteering) in long and middle-distance events, from 2005 to 2023, have been analyzed (sprint format events were excluded). Different categories from ten to twenty years of age for both sexes were included, and each category was divided into two years (1Y, 2Y) and two semesters (1S, 2S) within each year, creating four independent variables from the combination of year and semester (1Y1S, 1Y2S, 2Y1S, and 2Y2S). A total of 7,731 entries were examined, 4,318 were boys and 3,109 were girls. Descriptive statistics were analyzed for each variable, showing the mean and standard deviation. Normal distribution was confirmed for all variables (*p* > 0.05). Results showed a significant performance difference in favor of older orienteers in the youngest categories (U-10, M/F-12, M/F-14, and M-16), with these differences disappearing as age and performance increased (M/F-18 and M/F-20). These findings support the research hypothesis and align with other studies where age-related differences have also been found in other sports.

## Introduction

Orienteering is a sport that involves navigating through unfamiliar terrain using a map and compass to locate specific points in the shortest time possible. Orienteering can take place in various environments such as forests, urban areas or parks, making it a versatile and engaging outdoor activity for people of all ages. Participants must use their navigational skills to choose the best route between control points marked on the map by combining it with their displacement capacity. It combines physical exercise with mental challenges, requiring both physical fitness and strategic thinking.

The popularity and understanding of orienteering have been on the rise globally in recent years, which could potentially influence the emergence of Relative Age Effects (RAEs). Despite the scarcity of comprehensive studies that contribute to the enhancement and peak performance of orienteering athletes, emerging research aims to investigate some of the most crucial aspects of orienteering. For instance, the systematic review by Batista et al. (1) provides an exhaustive overview of studies from recent decades concerning the physiological and cognitive demands of orienteering. Subsequent studies have also focused on evaluating cognitive performance and its correlation with physical performance (2, 3). Others have explored the benefits of orienteering practice in preventing cognitive decline (4), addressed issues related to musculoskeletal injuries and training patterns (5–7), and examined the relationship between various physical abilities, motor coordination skills, and orienteering practice (8, 9). In the realm of nutrition and anthropometry, specific studies are beginning to surface (10). Additionally, investigations into technical errors in orienteering have been conducted (11). Moreover, research on navigation skills (12, 13) and mental rotation skills (14), although not directly specific to orienteering, have significant applicability. These studies underscore gender differences, and one of them (12) even discusses the potential impact of cultural activities (citing the example of orienteering in Nordic countries) on cognitive performance.

However, to identify and clarify the key factors of orienteering performance, the potential predictors of talent, and to determine the best methods of assessment, there is a clear limitation and controversy. Although there are studies, including some very recent ones, they are insufficient, suggesting a need to expand this line of research (15–19). This limitation primarily stems from the nature of orienteering and its conditions during the competition -different terrains, routes, height differences, or the interaction among runners during the race–making the monitorization of the performance a difficult task, with specific field test. Consequently, the results of the competition could be a key factor in tracking the sports talent in orienteering just as it is done in other similarly competitive nature sports (20).

Talent selection processes in orienteering primarily rely on performance in regional and national competitions, where athletes are evaluated for their navigation skills and physical endurance. Additionally, coaches play a crucial role in determining training opportunities, often prioritizing athletes who demonstrate superior performance at an early stage. The number of competitions per season varies, with a structured series of regional and national events allowing athletes to accumulate points and gain deliberate practice (21). Performance in these events often influences future competition levels, as stronger results lead to more advanced participation opportunities. Importantly, orienteering follows a cut-off date of January 1, meaning that athletes born early in the year may have a developmental advantage over those born later.

Accordingly, to form competitive groups within sports organizations, one fundamental criterion commonly employed is the year of birth. While this method is straightforward and often utilized in established systems like education, it poses certain drawbacks for the optimal nurturing of sporting talent. Nonetheless, for a competition to be effective, the performance of orienteers should be compared on an equal basis. An individual born in January is nearly twelve months older than someone born in December of the same year. Consequently, any physical and psychological advantages or disadvantages that may arise could be attributed to this age gap (22). The consequences attributed to those differences have been studied extensively as the Relative Age Effects -RAEs-, being more prominent in younger age groups (23, 24). Additionally, in orienteering, the competitive groups are defined with a range of two years (for children and teenagers) and therefore the aforementioned differences could increase significantly.

Understanding how RAEs influence sports performance requires acknowledging the complexity of identifying factors that contribute to talent development. The process of identifying such factors is inherently multifaceted (25). For instance, first-level influences—such as genetics, training, and physiological factors—play a direct role in shaping talent (26). However, secondary and indirect factors, like the RAE, can also significantly affect the opportunities and developmental pathways of young athletes (27). These secondary influences are particularly relevant in the context of orienteering, where competitive age groupings and environmental conditions may exacerbate disparities.

These consequences can be interpreted as a systematic discrimination and/or unequal opportunities for those individuals born shortly before the cut-off date of the competitive selection year (28). From another perspective, an orienteer might perceive a lack of sporting prowess or ability compared to their peers -within their competitive group- at a particular moment, potentially leading to negative feelings regarding their skills (29). This could result in a misguided belief that they are not achieving the same levels of success as others, diminishing their motivation in sports and increasing the possibilities of dropout (30, 31). As an example of this fact, one could cite scientific literature focusing on Athletics, which indicates that under-18 and under-20 athletes born in the first week of the year are about 2–3.5 times more likely to be included in the top-100 ranking than the athletes born in the last week of the year (28); some authors have investigated this phenomenon, referring to it as the Galatea effect in sport (32). The decisions made by coaches or scouts, as well as the criteria outlined in training programmes, may be influenced by short-term performance, prioritising maturation aspects over the innate potential of the orienteer. This approach can impede the development of long-term sporting talent (33), depriving vulnerable individuals of the deliberate experiences necessary to achieve expert performance (34).

The concept of constituent year effects refers to the impact of an athlete's birth year within a multi-year age category and its influence on competitive outcomes. Unlike the RAE, which examines disparities arising within a single-year age group, constituent year effects focuses on differences that emerge due to an athlete's birth position within a multi-year cohort. Research suggests that athletes born in the earlier years of a multi-year age category may have a competitive advantage over their younger counterparts due to increased physical and cognitive development during early stages of participation. For instance, studies on elite German youth basketball players indicate that the proportion of athletes per constituent year varies across age categories, with older individuals within the multi-year bands being overrepresented at higher competitive levels (35). Similarly, research on age-group effects in various sports suggests that constituent year effects prevalence can influence both individual development trajectories and overall team performance (36). Additionally, a longitudinal study on French top youth table tennis players found that birth quartiles were significantly associated with performance trajectories, particularly among male athletes under 18, highlighting the long-term impact of early relative age advantages within multi-year competitive structures (37). These findings imply that the structure of age-group classifications in sports can inadvertently benefit athletes born earlier in the designated age bands, potentially creating long-term disparities in skill acquisition, training opportunities, and career progression.

In consequence, it has been observed that RAEs influence coaches' decisions, favouring greater opportunities to add competitive hours (38). This data underscores the notion that older individuals — in the same year — have increased opportunities for skill acquisition within a competitive setting. Moreover, these elevated expectations from coaches or even family members would favour individuals with more advantageous RAEs, a phenomenon known as the Pygmalion effect in sport (32). Thus, the resulting consequences create a feedback loop that perpetuates advantages for those with more beneficial RAEs; this phenomenon is also explored in sports, termed the Matthew Effect (32), which necessitates mitigation from a professional standpoint.

Numerous comprehensive and recent scientific studies have explored the concept of Relative Age Effects (RAEs) in individual sports (39–46). However, when we narrow our focus to orienteering specifically, there is a scarcity of research linking RAEs to this sport. To our knowledge, only one recent study (47) has addressed RAEs in orienteering. Conducted in Sweden, this research presents an intriguing comparative analysis of the impact of RAEs on performance across various sports—Cross-country skiing, Alpine skiing, Athletics, Orienteering, Chess, and E-Sports- It also categorizes participants by age range, but does not consider competitive groups. Another related study (48), although not specific to orienteering and conducted within the context of soccer, associates RAEs with cognitive-attentional functioning. This aspect could be crucial for enhancing performance and identifying talent in orienteering. Despite these contributions, no study to date has demonstrated the relationship between relative age, competitive groupings, and orienteering with a broad sample and rigorous methodology. This gap in the literature sparks interest within the scientific community.

Once a comprehensive overview of the current state of research is provided, further investigation is warranted to ascertain how various variables influence talent development in young orienteers—factors such as age, gender, performance level, and experience—. Therefore, building upon the findings from the literature review mentioned earlier, the initial hypothesis posits that:

**Hypothesis (H1).** Older orienteers will show better performance than younger orienteers within the same competitive group, both girls and boys.

## Materials and methods

A transversal study design was used because this research concerned results from national events in Spain within the different youth categories. Thus, the overall results of the races were analysed. Therefore, the research design was based on an retrospective analysis without interference in the natural context of the events under study. Orienteering has experienced steady growth in Spain, particularly in youth categories, with an increasing number of participants each year. According to FEDO (Spanish Federation of Orienteering) reports, Spain hosts multiple national and regional events annually, with thousands of registered orienteers actively competing across different age groups. Despite being a specialized sport compared to traditional team sports, orienteering has gained recognition as an important discipline in outdoor and endurance sports.

### Sample

All data originated from the official results of the FEDO in long and middle distance events from 2005 (the first year in which data is available through electronic control systems) to 2023 were included, that is, 19 seasons. The races in sprint format (urban) were excluded from the analysis.

All the results analysed correspond to races belonging to the national league in young categories, included Under 10 (9–10 years old, boys and girls competing together). The categories or competitive groups included are (M = male, F = female): M/F12 (<12 years old), M/F14 (13–14 years old), M/F16 (15–16 years old), M/F18 (17–18 years old) and M/F20 (19–20 years old). A total of 7,731 entries were examined, 304 were boys and girls in U-10; 4,318 of which were boys and 3,109 were girls (Table 1), representing the whole context analysed (100%). In order to avoid bias in the research the same individual could register different entries for different seasons. However, those orienteers who competed in competition group above their chronological age were excluded from the analysis (*n* = 23). Thus, this registry of the data contributes to a greater understanding of the cases, due to the same orienteer being able to provide data related not only with its first year but also with its second year of the next season. FEDO was informed of the aim of the study and gave its consent for the publication of the data, anonymously, after signing a confidentiality agreement with the University of Alicante.

**Table 1:** Frequency of participation according to sex, competitive group and relative age semester.

Competitive group	Girls (*n* = 3,109)					Boys (*n* = 4,318)					U-10 (*n* = 304)
	F12	F14	F16	F18	F20	M12	M14	M16	M18	M20	
2Y1S	184	289	196	90	62	235	367	285	140	103	103
2Y2S	145	243	181	79	45	199	317	281	129	95	91
1Y1S	140	241	257	148	89	192	333	328	218	140	60
1Y2S	111	209	215	124	61	151	276	291	178	128	50

M/F 12 (< 12 years old), M/F 14 (13–14 years old), M/F 16 (15–16 years old), M/F 18 (17–18 years old) and M/F 20 (19–20 years old); 1Y1S, first year within competitive category and first relative-age semester; 1Y2S, first year in competitive group and second relative-age semester; 2Y1S, second year and first relative-age semester; 2Y2S, second year and semester.

### Procedure

Firstly, we identified both year (1Y or 2Y) within the competitive group and relative age semester (S1 = January–June; S2= July–December) as an independent variable for all the orienteers that participated, in at least one race, in the National Federation League of orienteering in Spain during the past 19 seasons 2005–2023 in the categories indicated above (Table 1). Therefore, four independent variables were used from the combination of the year and the semester: *1Y1S* = First season within competitive category and first relative-age semester; *1Y2S* = First season in competitive group and second relative-age semester; *2Y1S* = Second season and first relative-age semester; *2Y2S* = Second season and semester.

Secondly, the performance indicator—PI—was calculated in all the races. This indicator, used in other sports with the same monitoring and control difficulties (49), is calculated from the results in competition, which are obtained through a chip-timing system that allows to check an overall time for each individual with a high level of precision. PI is expressed from 0 to 100.00 points, with 100.00 being the score for the best time of the race and, therefore, the best performance. The rest of the orienteers' performances were calculated as a proportional part of that mentioned best time [PI = (Best Time × Personal Time^−1^) × 100]. An exception is set for the results in the Spanish championships, as the score for the best performance increases by 5% (105 points for the best performance).

Finally, a dependent variable was calculated from the PI as the average of all PI achieved by the individual over long and middle distance races during that season.

### Statistical analysis

The analysis of the data was carried out using SPSS® by IBM® software which was released in version 28 and with Microsoft Excel® for Mac in its 16.83 version. Descriptive statistics were analysed for each variable, showing the mean and standard deviation. In the different analyses, normality test was applied to the continuous variables (Shapiro–Wilk for *n* < 50 or Kolmogorov–Smirnov for *n* > 50). As a result, a normal distribution was shown for all variables (*p* > .05). In consequence, Anova 1-way and *post-hoc* test was applied. Since the homocedestacity criterion was not met, the statistical test *Games-Howell* was considered. Using Microsoft Excel software, the effect size was calculated (50). The effect size results (*ω*^2^) were classified into small results (0.01–0.06), medium results (>0.06–0.14), and large results (>0.14) (51). The level of significance was established in .05 in all cases except for the correction of Bonferroni (*p* < .008) in the *post-hoc* peer comparison.

## Results

**Figure 1:**
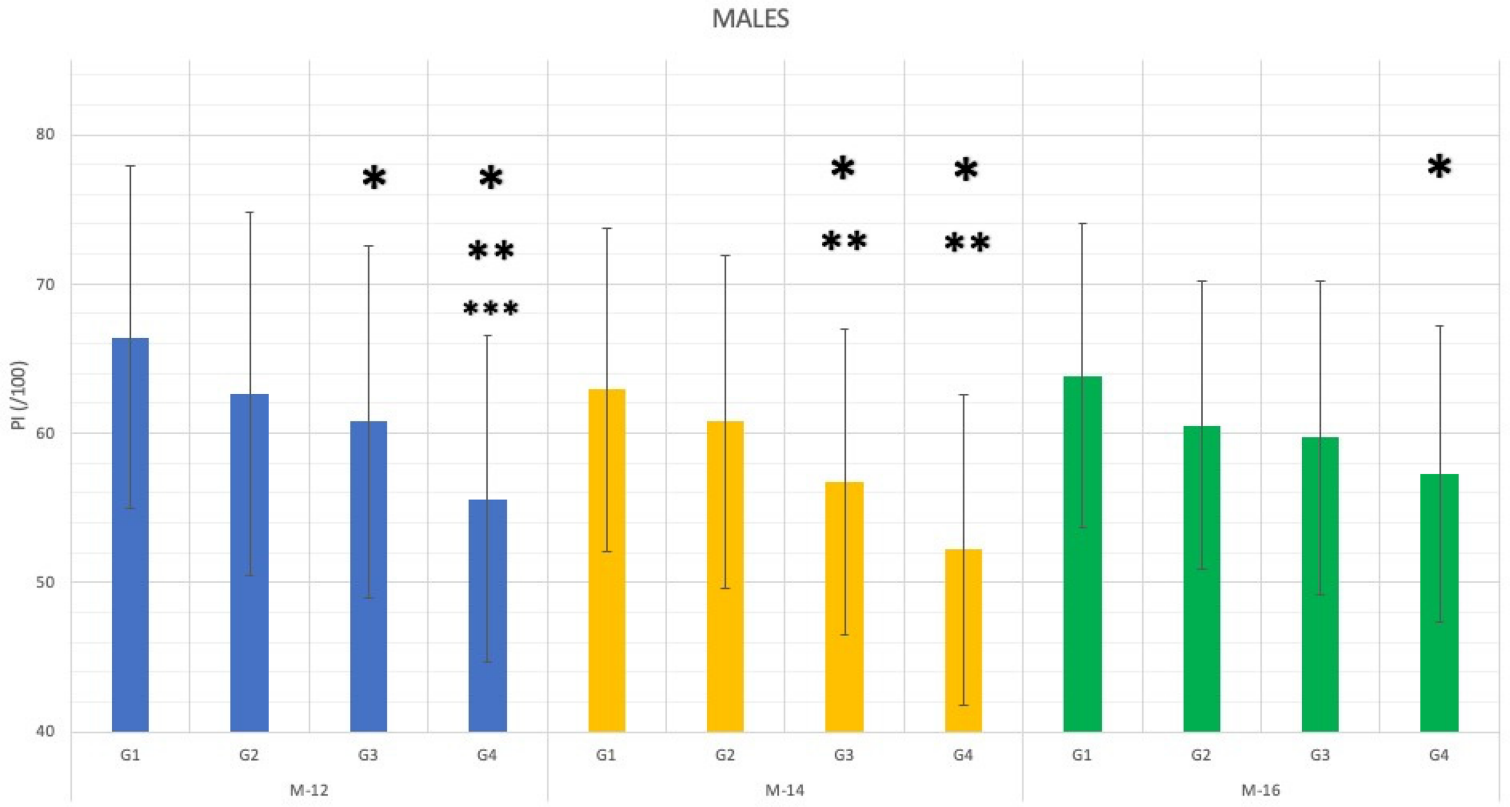
Bars graph representing the results of *post-hoc* games-howell test for the performance difference depending on the semester and year of birth of male children orienteers (*p < .0083 compared to G1; **p < .0083 compared to G2 and ***p < .0083 compared to G3).

**Figure 2:**
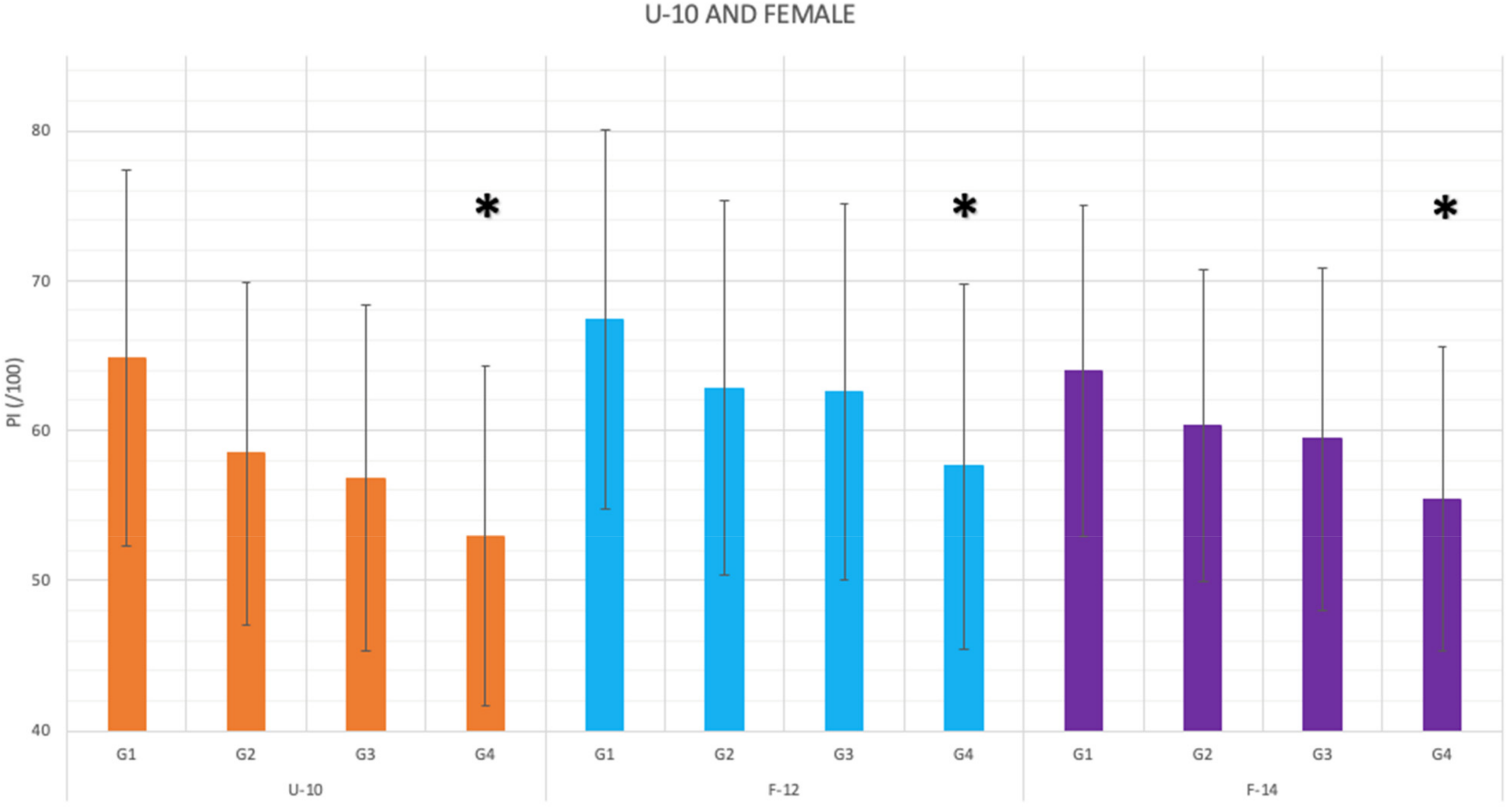
Bars graph representing the results of *post-hoc* games-howell test for the performance difference depending on the semester and year of birth of under-10 and female children orienteers (*p < .0083 compared to G1).

First, based on the relative age semester (Table 2), it was observed that for each competitive group, the best results were obtained by both, male and female orienteers born in S1 (January–June). Secondly, as a result, Anova 1-way test results indicated that, based on the performance differences depending on the specific year and semester of birth (Table 2), statistical differences (*p* < .001) were observed in the youngest categories, this is, M-12, M-14 and M-16 (Figure 1) and U-10, F-12 and F-14 (Figure 2).

**Table 2:** ANOVA-1 way test for performance in orienteering races according to the different groups (average ± standard deviation).

Sex	Competitive group	Older → Younger				F-ANOVA (sig.)	ES (*ω*^2^)	*post-hoc*: Games-Howell (Effect Size, *ω*^2^)										
		G1	G2	G3	G4			1vs2	1vs3 Sig. (ES)		1vs4 Sig. (ES)		2vs3 Sig. (ES)		2vs4 Sig. (ES)		3vs4 Sig. (ES)	
Mixed	U-10	64.8 ± 17.2	58.4 ± 19.1	56.8 ± 17.6	52.9 ± 18.6	5,615*	(.044)	—	—		.002	(.043)	—		—		—	
Female	F-12	67.3 ± 16.9	62.8 ± 18.0	62.5 ± 15.1	57.6 ± 17.2	7,912*	(.034)	—	—		<.001	(.037)	—		—		—	
	F-14	63.9 ± 16.2	60.3 ± 17.1	59.4 ± 17.3	55.4 ± 16.8	10,595*	(.028)	—	—		<.001	(.030)	—		—		—	
	F-16	65.4 ± 16.7	65.1 ± 14.4	63.8 ± 15.8	63.0 ± 15.2	1,097	—	—	—		—		—		—		—	
	F-18	67.7 ± 17.1	64.2 ± 13.7	66.9 ± 15.9	63.7 ± 14.6	1,737	—	—	—		—		—		—		—	
	F-20	69.0 ± 15.7	69.6 ± 15.9	68.0 ± 16.2	66.0 ± 17.4	0,525	—	—	—		—		—		—		—	
Male	M-12	66.4 ± 18.8	62.6 ± 18.1	60.7 ± 17.4	55.6 ± 17.7	11,316*	(.038)	—	.008	(.012)	<.001	(.039)	—		.002	(.015)	—	
	M-14	62.9 ± 17.2	60.7 ± 15.7	56.7 ± 16.4	52.2 ± 16.4	25,390*	(.054)	—	<.001	(.017)	<.001	(.048)	.008	(.006)	<.001	(.028)	.004	(.008)
	M-16	63.8 ± 16.0	60.5 ± 15.7	59.7 ± 16.3	57.2 ± 14.8	7,992*	(.019)	—	—		<.001	(.020)	—		—		—	
	M-18	64.2 ± 15.6	62.3 ± 15.0	65.2 ± 15.2	62.7 ± 14.5	1,355	—	—	—		—		—		—		—	
	M-20	70.2 ± 17.8	68.2 ± 16.4	67.6 ± 17.0	65.7 ± 15.2	1,449	—	—	—		—		—		—		—	

ES = Effect Size (*ω*^2^); M/F 12 (<12 years old), M/F 14 (13–14 years old), M/F 16 (15–16 years old), M/F 18 (17–18 years old) and M/F 20 (19–20 years old); G1, 2Y1S (second year and first relative-age semester); G2, 2Y2S (second year and second relative-age semester); G3, 1Y1S (first year within competitive category and first relative-age semester); G4, 1Y2S (first year in competitive group and second relative-age semester).

*p < .001.

The Games-Howell statisticians of the *post-hoc* analysis (considering Bonferroni correction to avoid type II error; *p* < .0083) showed differences in favour of the older group (G1 = 2Y1S) compared to the younger group (G4 = 1Y2S) in all competitive groups that showed differences in Anova (U-10, M/F-12, M/F-14 and M-16). Specifically in males, M-14 presented differences among all its competitive groups except for G1 over the second largest (G2 = 2Y2S). Differences were also shown between G1 over the second younger group (G3 = 1Y1S) and G2 Vs. G4, in M-12 (Table 2, *post-hoc*).

## Discussion

The main aim of this research was to investigate possible differences in performance among young orienteers in early orienteering, considering both the year of birth and the relative age semester. The most relevant result of this research showed a significant performance difference in favor of older orienteers within the same competitive group in the youngest categories (U-10, M/F-12, M/F-14 and M-16), disappearing these differences as age and performance increases (47, 52). These findings support the research hypothesis put forward in this study (H1 is accepted).

While this investigation focuses on the impact of competitive grouping based on chronological age criteria rather than directly examining RAEs, the findings align with those of other studies where differences have been found in other sports (23, 47, 49). Evidently, both RAEs and competitive grouping, as documented in previous literature (22, 29), play significant roles in shaping the opportunities for athletes to attain the highest levels of sports talent development (32). These authors proposed a socio-psychological framework to understand the broader implications of RAEs, highlighting three interrelated mechanisms: the Pygmalion effect, where greater expectations are placed on relatively older athletes; the Matthew effect, where initial advantages compound over time; and the Galatea effect, where self-perceptions of competence influence performance. These mechanisms suggest that RAEs not only affect physical and technical development but also the social and psychological environment, amplifying disparities between older and younger athletes in the same group. Addressing these underlying mechanisms is crucial to creating equitable talent pathways in orienteering and other sports.

It is true that an athlete who is in his first year (the youngest, 1Y), the following year moves to the second year (2Y), and the cycle gradually balances out from one competitive group to another. However, if the orienteer is born in the second half of the year (and he is under-16 or she is under-14), meaning between July and December, they will consistently encounter significant differences as shown in this study, regardless of gender, thus carrying a heavier burden for their optimal development and potential performance in the future. While statistical differences attributed to competitive grouping were not detected for rest of competitive groups (F-16, M/F-18, M/F-20) in this study, it is noteworthy that, in all categories, 100% of the top performances were achieved by orienteers born in the first semester of the year, as reported in other research (29, 47, 48, 53, 54). Just as it was applied in other studies (55), further studies are needed to determine how the results shown in this research impact the selection processes of national and regional coaches and scouts in orienteering.

Consistent with the preceding paragraph, a notable impact on performance discrepancy within competitive groups was observed, particularly among the youngest cohort of orienteers, aged between 13 and 14, as supported by other recent studies (56). This underscores the significance of factoring in both RAEs and maturation status (57) since this difference could increase by up to four years. Being comparatively older and concurrently at a more advanced stage of maturation confers a significant advantage in performance-related attributes. Furthermore, Hancock et al. (32) emphasized that relatively older athletes often receive more resources, such as coaching attention and competitive opportunities, which perpetuate the RAEs cycle. This allocation of resources may inadvertently marginalize younger athletes, leading to a self-fulfilling prophecy where perceived underperformance among the relatively younger persists across their development. Although RAEs are generally less pronounced in female athletes, some research has identified small effects, particularly in team sports (23, 28, 29, 55, 56, 58, 59), this study cannot support this statement. However, when analysing the results of boys and girls, it is observed that significant differences in the F-16 group disappear in girls, while in boys they continue in M-16 that later disappear, possibly indicating a diminishing impact of growth and maturation processes on sports participation, particularly noticeable in women, occurring two years earlier than in men (60). Therefore, RAEs also affects the female sports context in orienteering, as demonstrated by the review and meta-analysis conducted by Smith et al. (52).

In alignment with the findings of our research, it is also worth highlighting the study by Jakobsson et al. (47), which bears a striking resemblance to the present research. These authors gathered data from athletes born between 1922 and 2015, spanning various sports, including orienteering (*n* = 41,164). The data, in this case divided into four-month periods (tercils: T1, T2, T3), unveiled a significant bias in the distribution of birth dates across all sports, both sexes, and most age groups. In relation to its results specific to orienteering sport, RAEs were identified across various age groups: ≤8, 11–15, 16–20, 21–39, 40–59, and ≤60 years, the effect sizes (*V*) were 0.25, 0.19, 0.11, 0.17, 0.06, 0.13, and 0.16, respectively, and notably, significant RAEs were evident in all age groups, including boys and girls up to 5 years old (*p* < .001, *V* = 0.37). However, in adults over 60 years, the distribution was nearly equal (approximately 33% per tertile), and an inversed RAE was noticeable in both sexes (*p* < 0.05, *V* = 0.14, ratio T3 = 1.06 for males; *p* < 0.05, *V* = 0.19, ratio T3 = 1.09 for females). Following their study, when considering the entire analyzed sample, RAEs were consistently observed in the majority of individual sports in Sweden, including those that are physically demanding and those requiring cognitive skills. Their findings, which mirror those of the current study, suggest that in most sports, children born earlier often outperform and outrank their later-born counterparts. However, this trend is not evident among adult athletes, where no discernible correlation between birth date and performance exists. Jakobsson et al. (47) concluded that neglecting to address the issue of relative age early on could result in a restricted and arbitrary selection of elite adult athletes, disproportionately favoring those born earlier in the selection year while prematurely excluding potentially talented but younger athletes. Additionally, the lack of intervention could negatively impact public health, as early dropout from organized sports due to RAE disadvantages may lead to lower levels of lifelong physical activity, increasing the risk of sedentary lifestyles and associated health problems such as obesity and cardiovascular diseases. This lends credence to the idea that RAEs and competitive grouping significantly shape the opportunities available for athletes to excel in sports talent development. Consequently, these findings emphasize the importance of considering both RAEs and maturation status when selecting and developing young athletes in orienteering and other sports. These authors also underscore the need for additional studies to comprehend how these findings influence the selection processes employed by national and regional coaches in orienteering and other sports.

Considering the unique characteristics of orienteering sport and the significant role cognitive performance may play, the results obtained by Huertas et al. (48) in a team sport context can be related to our study objectives. The researchers compared attentional functioning, anthropometry, physical fitness, and game intelligence across two age groups (U10 vs. U12) and four birth trimesters (BQ1–BQ4). Their findings revealed a statistically significant RAE (*p* < .001), with approximately 50% of the participants born in the first trimester and 75% in the first half of the year. However, they found no age effect on the functioning of attentional networks. This lack of age effect was attributed to the maturation of attentional networks, which typically reach peak development around nine years of age, and the quality of practice. They noted that athletes in elite teams, due to superior training and competition experiences, have similar daily opportunities for comprehensive development in physical, technical, tactical, and perceptual-cognitive aspects. They further suggested that despite individual baseline differences, elite athletes tend to achieve comparable cognitive benefits. Another possibility they proposed is that relatively younger players, unable to solve game situations by leveraging superior physical abilities, are compelled to enhance their game intelligence and, consequently, their perceptual-cognitive abilities. These insights could be applied to the training and selection of young orienteering athletes. The gap between maturation status and biological age, particularly in physical differences, could be compensated in orienteering learning and training with a focus on technical-tactical and perceptual-cognitive aspects. Additionally, selection or talent identification tests that include cognitive performance assessment, especially for younger competitive groups, could help mitigate the effect of RAEs. Nevertheless, further research is needed on the impact of RAEs and competitive groupings on cognitive performance.

The present study is not without limitations. While the performance indicator used, namely PI, is deemed suitable for assessing performance, especially for tracking purposes, this variable is limited to winner time. Although this approach is better to using the average time of all orienteers (29), winner time is inherently contingent upon the performance of the individual athlete. As outlined in the method section, the dependent variable (individual performance) was calculated as the average value of the performance indicator across competitions for each orienteer within a season. Notably, some orienteers participate in two races, while others partake in four. Additionally, the use of a cross-sectional design and the categorization by birth semesters may limit the depth of the analysis, as this approach may not fully capture seasonal variations and other temporal factors that could influence performance and age distribution. However, this design is common in the relative age effect literature and provides a valuable snapshot for identifying trends and associations at a given point in time. Future studies could benefit from longitudinal approaches to overcome these limitations and examine corrective adjustments, as suggested in prior work (23), offering a more comprehensive understanding of the impact of relative age on orienteering performance over time.

## Conclusion

The findings of this study confirm that younger orienteers tend to perform worse within their competitive groups, while older counterparts show enhanced performance. The complex process of talent development is influenced by primary factors like genetics and training (26) and secondary factors such as RAEs (27). This study highlights the impact of competitive grouping in orienteering, particularly disadvantaging athletes born in the second half of the year.

To promote fairer competition, adjustments in competitive grouping should be considered, especially for young orienteers approaching peak height velocity (60). One potential solution is a refined ranking system within races, segmenting classifications by birth period (e.g., 1Y1S; 1Y2S; 2Y1S; 2Y2S). Alternatively, bio-banding or classification based on biological age (48, 61) could be explored, though adaptation to orienteering would require defining and measuring technical-tactical and cognitive skills. Additionally, reserving spots for younger orienteers may help reduce dropout rates (25, 62, 63) and prevent the loss of potential talent.

## Data Availability

Publicly available datasets were analyzed in this study. This data can be found here: The competition results used in our study are publicly available and were obtained from the following page: https://www.fedo.org/web/archivo-resultados-o-pie. The data regarding the birth dates of federated athletes were provided by the Spanish Orienteering Federation (Federación Española de Orientación, FEDO). These data are not publicly accessible. As such, there are legal restrictions on sharing this dataset, as imposed by the Spanish Orienteering Federation. Designated contact for data inquiries: secretaria.fedo@gmail.com.
